# Supplementary material for: Carbon footprint assessment and crashworthiness evaluation of alloy steel W-beam guardrails
Source: PLoS One. 2026 Apr 17;21(4):e0347115. doi: 10.1371/journal.pone.0347115 (PMC13089726; doi:10.1371/journal.pone.0347115)
Supplement: Attachment — Submitted filename: 1 Response to Reviewers.docx [file pone.0347115.s005.docx]

****Manuscript ID:**** PONE-D-25-60856

****Title:**** Carbon Footprint Assessment and Crashworthiness Evaluation of Alloy Steel W-beam Guardrails

Dear Reviewers,

Thank you for the opportunity to revise and resubmit our manuscript. We sincerely appreciate the editor’s consideration and the constructive, detailed feedback provided by all three reviewers. Their comments have been invaluable in strengthening the manuscript's clarity, depth, and overall quality.

We have carefully addressed every point raised. The revisions are extensive and have significantly enhanced the technical rigor, methodological transparency, and academic presentation of the work. Below, we provide a point-by-point response to each reviewer's comments. All changes in the manuscript are highlighted in the “Revised Manuscript with Track Changes” file.

Below, we provide a detailed, point-by-point response to each comment.

### ****Response to Reviewer #1****

**General Comment:** The reviewer noted a lack of innovation in experimental design and result analysis, with insufficient detail in methods and superficial data analysis.

**Response:** We thank the reviewer for this critical assessment. To address these fundamental concerns about methodological depth and analytical rigor, we have undertaken major revisions throughout the manuscript, transforming it from a primarily descriptive report into a more analytical and framework-driven study.

**Enhanced Methodological Detail and Innovation:** We have significantly expanded the Materials and Methods (Section 2) to enhance reproducibility and transparency. This includes:

- Specifying the alloy steel grade as 700L (Section 2.1).
- Detailing the parallel experimental framework with explicit Experimental (alloy steel) and Control (Q235 steel) groups, including their specific structural dimensions optimized for lightweighting (Section 2.1).
- Providing comprehensive parameters for the LCA model, including the eFootprint software configuration (version, assessment standard, allocation rules, cut-off threshold) and specific emission factors from the CLCD 2024 database (e.g., grid electricity: 0.581 tCO₂e/MWh) (Section 2.2).
- Listing the specific sensor models, measurement ranges, accuracies, and data acquisition parameters (sampling frequency, filtering method per ISO 6487/SAE J211/1) used in the crash tests (Section 2.4).

**Deepened Result Analysis and Theoretical Context:** The Results and Discussion sections have been completely restructured and greatly expanded to move beyond simple data reporting.

- A new Section 3.1.3 "Key Parameter Sensitivity Analysis" with Table 7 has been added. This analysis quantifies the influence of core parameters (hot metal, electricity, etc.) on the carbon footprint, directly addressing the need for more sophisticated data analysis and providing insights for future optimization.
- The safety results (Table 8) now explicitly include parallel data for the Q235 control group, allowing for a direct, like-for-like comparison and strengthening the evidence for the alloy steel's performance advantages.
- The Introduction (Section 1) has been completely rewritten. It now provides a synthesized, critical review of international and domestic literature, clearly identifies three specific research gaps, and states four focused objectives that define this study's contribution—moving away from a simple list of citations.
- The Discussion (Section 4) has been reorganized into logical subsections (4.1 Carbon Reduction Mechanism, 4.2 Safety Performance Improvement, 4.3 Synergistic Mechanism, 4.4 Cost-Benefit Analysis). The language has been refined to be more academic and analytical, eliminating repetition and report-style phrasing. The new Section 4.4 provides a quantitative cost-benefit analysis, directly addressing engineering practicality.

We believe these comprehensive additions directly respond to the reviewer's concerns by demonstrating greater innovation in the integrative analytical framework and providing the deep, quantitative analysis of results that was previously lacking.

### ****Response to Reviewer #2****

We thank Reviewer #2 for the supportive assessment and valuable specific suggestions.

**Comment 1 (Language):** Correction of the typographical error: “EPcisthe” to “EPc is the”.

**Response:** Thank you for spotting this. The error has been corrected in the formula description in Section 2.2.

**Comment 2 (Methods Detail):**

(1) **Describe sensor types and data acquisition.**

**Response:** As detailed in our response to Reviewer #1, this information has been added to Section 2.4. We now list the accelerometer (PCB 356A15), velocity sensor (Kistler 6045A), data acquisition system (NI cDAQ-9178), and high-speed camera (Phantom V2512) models and key specifications, including the 100 Hz low-pass filtering protocol.

(2) **Mention the commercial/in-house designation of the alloy steel.**

**Response:** The alloy steel is now identified by its industrial grade 700L in Section 2.1.

**Comment 3 (Data Availability Statement):** Revise to comply with PLOS ONE policy, as the previous statement was contradictory and overly restrictive.

**Response:** We sincerely apologize for the previous inconsistency. The Data Availability Statement has been revised to accurately and compliantly reflect the situation:

The processed acceleration time-history data for the crash tests are provided in the Supporting Information files (S1-S4 Files).”

We have also uploaded the acceleration data files as Supporting Information (S1-S4 Files).

### ****Response to Reviewer #3****

We are grateful to Reviewer #3 for the exceptionally thorough and insightful critique, which has driven the most substantial improvements to the manuscript.

**Comment 1 (Introduction & Literature):** The introduction simply listed papers; it should acknowledge existing research and clearly define the innovation point.

**Response:** The Introduction has been completely rewritten (Section 1). We have replaced the long list of citations with a structured, thematic literature review that:

- 1. Critically surveys international advances in materials, low-carbon processes, and LCA.
- 2. Reviews domestic progress while clearly highlighting persistent gaps (e.g., lack of systemic coupling analysis).
- 3. Explicitly articulates three core research gaps and outlines four specific objectives of this study that address them, thereby sharply defining our contribution and innovation.

**Comment 2 (PSE Metric Justification):** Provide supporting references for the use of PSE in guardrail safety evaluation.

**Response:** We have added the necessary theoretical and empirical support in Section 2.1. The text now states: “Existing research, through full-scale vehicle crash tests, has confirmed that when guardrail steel achieves a tensile strength above 700 MPa while maintaining an elongation after fracture of over 20%, it can absorb collision energy more fully through enhanced plastic deformation, significantly improving the protective stability of the guardrail [13,15].” References 13 (Liu, 2021) and the newly added 19 (Yang et al., 2022 on crashworthiness optimization considering strength-ductility balance) provide the required foundation.

**Comment 3 (Significance of Deflection Reduction):** Clarify the engineering significance of the 3.2% reduction and compare to code limits.

**Response:** We have refined the narrative to focus on the more meaningful finding: the alloy steel guardrail met all SB-level safety requirements (Table 8) while achieving a 25% and 50% reduction in beam and post thickness, respectively. The discussion in Sections 3.2 and 4.2 now emphasizes that the superior material properties (high PSE) enabled this lightweight design without compromising safety, which is the key engineering insight, rather than the minor percentage difference in a single metric.

**Comment 4 (Q235 Data Source & Parallel Test Data):** Specify the source of Q235 data and present corresponding data charts.

**Response:** This has been clarified and addressed:

- Source: Section 2.1 now explicitly establishes the parallel experimental framework, and Section 2.3 clarifies: “the data for the Q235 steel guardrail... are derived from a parallel experiment conducted at the same production scale. Its production conditions... were kept consistent...”
- Data Presentation: Table 8 has been expanded to include a dedicated “Control Group (Q235 Steel) Results” column, providing the comparative crash test data. The acceleration data for the Q235 guardrail (small car) are provided in the new Supporting Information files (S3 & S4 Files).

**Comment 5 (LCA Sensitivity Analysis): Sensitivity of key parameters was not assessed.**

**Response:** A new Section 3.1.3 and Table 7 have been added, presenting a one-factor-at-a-time sensitivity analysis for five key parameters (hot metal, electricity, etc.). This analysis identifies hot metal input as the most sensitive parameter and discusses implications for carbon footprint optimization.

**Comment 6 (Acceleration Data Filtering):** Specify the filtering method used in Figure/analysis.

**Response:** The data processing protocol is now clearly stated in Section 2.4: “The acquisition and processing... strictly adhered to... ISO 6487 and SAE J211/1. For analysis, the raw data was digitally filtered using a low-pass filter with a cut-off frequency of 100 Hz to attenuate high-frequency noise.”

**Comment 7 (Discussion/Conclusions Language):** Excessive repetition and engineering report-style language; needs academic editing.

**Response:** The Discussion (Section 4) and Conclusions (Section 5) have been entirely rewritten. The discussion is now logically structured, analytical, and free of repetition. The conclusions are concise, summarizing the three main findings without simply re-stating the discussion. The language throughout has been elevated to a more formal academic standard.

**Comment 8 (Cost-Benefit Analysis):** Include a brief cost-benefit analysis.

**Response:** A new Section 4.4 “Cost-Benefit Analysis” has been added. It provides a comparative analysis of initial and life-cycle costs (LCC), quantifying benefits from lightweighting (30-34% steel reduction), extended service life (15 to 25 years), reduced maintenance, and carbon asset value. It concludes that the alloy steel guardrail has a 13.8% lower LCC per kilometer, demonstrating its techno-economic viability.

### ****Summary****

We believe the revised manuscript has been fundamentally improved in direct response to all reviewer comments. Key enhancements include:

- A completely restructured and critical Introduction.
- Greatly expanded methodological detail for full reproducibility.
- A new sensitivity analysis for the LCA.
- Explicit parallel experimental data and comparison.
- A new, quantitative cost-benefit analysis.
- Refined, academic language throughout the Discussion and Conclusions.
- A compliant Data Availability Statement and provision of supporting data files.

We are confident that the manuscript now meets the high standards of PLOS ONE and effectively presents a novel, systematic, and data-rich evaluation of the synergistic safety and carbon reduction potential of advanced alloy steel guardrails.

Thank you again for your consideration.

Sincerely,

Shuai Gong, on behalf of all co-authors.

### ****To the Academic Editor:****

In addition to the point-by-point responses below, we wish to highlight that the major revisions undertaken have directly addressed the core concerns reflected in the reviewers’ summary assessments:

- Technical Soundness & Data Support (Reviewer #1: ‘Partly’):By expanding the methodological detail, adding sensitivity analysis, providing parallel experimental data for the control group, and deepening the discussion of mechanisms, we have substantially strengthened the technical rigor and the evidence linking our data to the conclusions. We believe the manuscript now firmly meets the criteria for technical soundness.
- Statistical/Rigorous Analysis (Reviewer #1: ‘No’):The addition of the sensitivity analysis (Section 3.1.3, Table 6) provides a quantitative assessment of parameter influence. Furthermore, the explicit description of test replicates (material samples in Table 2), controlled comparative experiments, and standardized data processing protocols (e.g., filtering in Section 2.4) collectively enhance the analytical rigor of the study.
- Data Availability (All Reviewers: ‘No’):We have revised the Data Availability Statement to accurately reflect the specific restrictions (proprietary industrial foreground data) and the pathway for qualified access, bringing it into compliance with PLOS ONE’s policy. The background LCA data from the public CLCD database is fully cited.

We are confident that these comprehensive revisions have resolved the issues underlying the reviewers’ initial summary judgments, and the manuscript is now much stronger and fully ready for publication.

Sincerely,

Shuai Gong, on behalf of all co-authors
